# Integrated Multichip Analysis and WGCNA Identify Potential Diagnostic Markers in the Pathogenesis of ST-Elevation Myocardial Infarction

**DOI:** 10.1155/2022/7343412

**Published:** 2022-04-07

**Authors:** Yingliang Liang, Wandang Wang, Qiufang Huang, Hui Chen

**Affiliations:** Department of Clinical Laboratory, Xiaolan People's Hospital of Zhongshan, Zhongshan, Guangdong, China

## Abstract

**Background:**

ST-elevation myocardial infarction (STEMI) is a myocardial infarction (MI) with ST-segment exaltation of electrocardiogram (ECG) caused by vascular occlusion of the epicardium. However, the diagnostic markers of STEMI remain little.

**Methods:**

STEMI raw microarray data are acquired from the Gene Expression Omnibus (GEO) database. Based on GSE60993 and GSE61144, differentially expressed genes (DEGs) are verified via R software, and key modules associated with pathological state of STEMI are verified by weighted correlation network analysis (WGCNA). Take the intersection gene of key module and DEGs to perform the pathway enrichment analyses by Gene Ontology (GO) and Kyoto Encyclopedia of Genes and Genomes (KEGG). Construct the protein-protein interaction (PPI) network by Cytoscape. Then, select and identify the diagnostic biomarkers of STEMI by least absolute shrinkage and selection operator (LASSO) logistic regression and support vector machine-recursive feature elimination (SVM-RFE) algorithms. Finally, assess the infiltration of immune cells of STEMI by CIBERSORT and analyze the correlation between diagnostic markers and infiltrating immune cells.

**Results:**

We get 710 DEGs in the STEMI group and 376 genes associated with STEMI in blue module. 92 intersection genes were concentrated in 30 GO terms and 2 KEGG pathways. 28 hub genes involved in the development of STEMI. Moreover, upregulated ALOX5AP (AUC = 1.00) and BST1 (AUC = 1.00) are confirmed as diagnostic markers of STEMI. CD8+T cells, regulatory T (Treg) cells, resting natural killer (NK) cells, M0 macrophages, resting mast cells, and neutrophils are related to the procession of STEMI. Moreover, ALOX5AP and BST1 are positively related to resting NK cells, M0 macrophages, and neutrophils, while ALOX5AP and BST1 are negatively related to CD8+ T cells, Treg cells, and resting mast cells.

**Conclusion:**

ALOX5AP and BST1 may be the diagnostic markers of STEMI. Immune cell infiltration plays a key role in the development of STEMI.

## 1. Introduction

ST-elevation myocardial infarction (STEMI), one type of MI diseases, is the main cause of human death [1]. Although mortality declines due to primary percutaneous coronary intervention (PCI) combined with modern antithrombotic pharmacologic therapy, the heart failure is still a challenge for survivors [2]. STEMI results in severe or complete blockage of the coronary artery [3, 4]. Currently, the routine diagnosis of STEMI is usually based on invasive approaches (myocardial blush grade, intracoronary physiology, and resistive reserve ratio) and noninvasive approaches (CMR imaging). However, the early diagnosis of STEMI is impossible [5]. Therefore, screening the biomarkers of STEMI patients is important to improve the prognosis of STEMI.

Recently, many studies find that immune cell infiltration is related to the pathological progression of STEMI. For instance, increasing the apoptosis of lymphocytes apoptosis and proinflammatory Th1 lymphocytes infiltration in the heart is shown in STEMI patients with PCI treatment [6]. STEMI heart exhibits an increase of immune cell infiltration, resulting in cardiomyocyte apoptosis and cardiac dysfunction [7]. Cell type identification by estimating relative subsets of RNA transcripts (CIBERSORT), an analysis tool, is used to assess the immune cells and obtain various immune cell ratios from RNA-seq data of samples [8, 9]. Analysis of immune cell infiltration in multiple diseases such as cancer [10], congenital heart disease [11], and systemic lupus erythematosus [12] has been widely used. However, the research to analyze immune cell infiltration of STEMI by CIBERSORT is little.

In our study, STEMI raw microarray data are acquired from the Gene Expression Omnibus (GEO) database, and differentially expressed genes (DEGs) are screened. Screen and confirm the diagnostic markers by machine learning algorithms. Subsequently, analyze the difference in immune cells infiltration between the STEMI group and normal group by CIBERSORT. Finally, verify the connection between diagnostic markers and infiltrating immune cells in STEMI.

## 2. Materials and Methods

### 2.1. Data Download

Get the expression profile datasets GSE60993 and GSE61144 of STEMI from the GEO database.

### 2.2. Data Preprocess and DEGs Screen

Merge the GSE60993 and GSE61144 gene expression matrices and use the “sva” package to remove differences between GSE60993 and GSE61144. Picture the effect of removing differences between GSE60993 and GSE61144 by quantile-quantile chart (Q-Q chart). Demonstrate the effect of batch correction by a two-dimensional PCA cluster chart. DEGs are filtered through the “limma” package, and draw the volcano map of DEGs by the “ggplot2” package and heat map of DEGs by the “pheatmap” package. DEGs with *p* < 0.05 and |log2FC| >1 are considered statistically significant.

### 2.3. WGCNA

Construct the coexpression network by WGNCA package. Remove the abnormal samples to ensure the network construction is credible. Then, set the soft threshold power. The key module with the high correlation with STEMI is identified.

### 2.4. Functional Correlation Analyze and PPI Network Construct

Taking the intersection of the key module gene set obtained by WGCNA and DEGs, 92 intersection genes are obtained. Then, the GO enrichment analysis (FDR < 0.05) and KEGG enrichment analysis (FDR < 0.05) of 92 intersection genes are performed by R package “clusterProfiler.” Furthermore, construct the PPI network of 92 intersection genes via the STRING and visualize by the Cytoscape. Minimum required interaction score ≥0.4 is considered statistically significant, and the hub genes in PPI network are constructed.

### 2.5. Screen and Verify Diagnostic Markers

Screen diagnostic markers of STEMI by LASSO and SVM-RFE. Then, verify the diagnostic biomarkers of STEMI via “e1071” package. Finally, combine the genes verified by LASSO or SVM-RFE algorithms to study.

### 2.6. Evaluate the Immune Cell Infiltration

Analyze the immune infiltration of STEMI and normal control samples by R package “CIBERSORT” to get the distribution of 22 immune cells in the STEMI group with a *p* value <0.05. Remove the three types of nonexpressing immune cells in the sample, and the box plot is used to compare the immune cells of STEMI and normal samples by box plot.

### 2.7. Analyze the Connection between Diagnostic Markers and Infiltrating Immune Cells of STEMI

Analyze the connection between diagnostic markers and infiltrating immune cells by “ggstatsplot” package and visualize the results by “ggplot2” package.

## 3. Results

### 3.1. Collect Data and Screen DEGs

**Figure 1:**
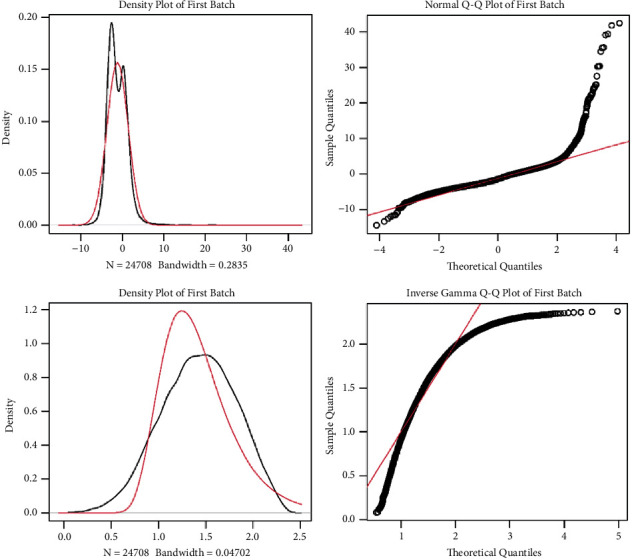
Remove the interbatch difference of GSE60993 and GSE61144 datasets by the Q-Q plot.

**Figure 2:**
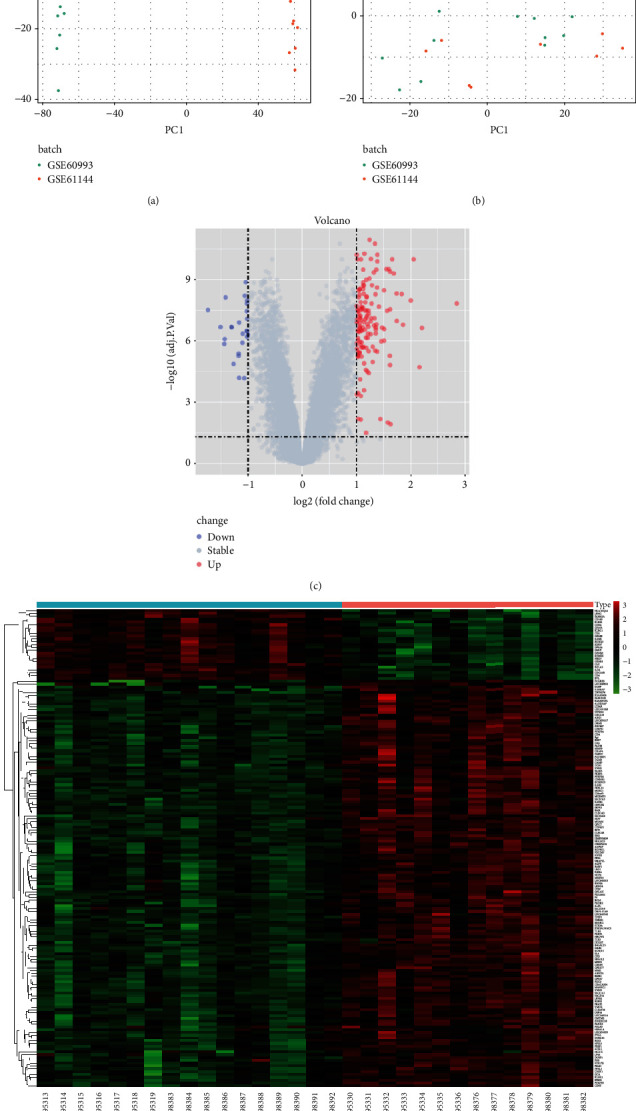
Screen the DEGs. (a)-(b) Two-dimensional PCA cluster plot of the GSE60993 and GSE61144 datasets before and after sample correction. (c) Volcano map of DEGs of the STEMI group. (d) Heat map of the DEGs STEMI group.

First, merge the datasets of GSE60993 and GSE61144, remove the interbatch difference of the gene expression data, and show the result by the Q-Q plot (Figure 1). Then, normalize and process the merged gene expression matrix, and the result shows in a two-dimensional PCA cluster diagram before and after normalization (Figures 2(a) and 2(b)). After preprocessing the data, extract 710 DEGs from the gene expression data of STEMI samples (Figures 2(c) and 2(d)).

### 3.2. Construct a Weighted Coexpression Network and Identify Key Modules

**Figure 3:**
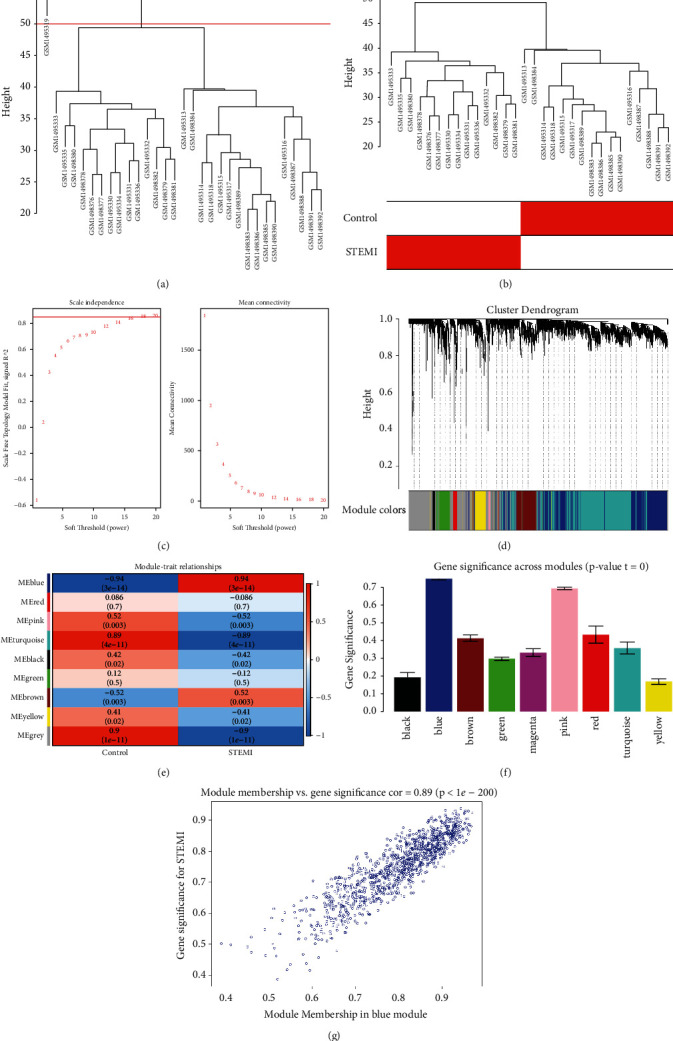
WGCNA. (a) Clustered sample. (b) Sample dendrogram and trait heatmap. (c) Analyzed network topology for various soft thresholding powers. (d) Clustered dendrogram of genes. (e) Heatmap of the module-trait relationships. (f) Gene significance across modules. (g) Blue module.

First, we cluster the samples and set the height cutoff value at 50, and one sample is excluded in our analysis (Figure 3(a)). Then, a soft threshold power with a scale-free *R*^2^ about 0.9 and a slope about 1 is picked. To cluster splitting, setting the soft thresholding power at 18 and the minimum module size at 30, 9 gene coexpression modules are constructed (Figures 3(b)–3(d)). Based on the criteria that cor ≥0.90, *p* < 0.001, blue module is confirmed as key module to study (Figures 3(e) and 3(f)). According to GS > 0.8 and MM > 0.8, the key genes of the blue module are screened, and 376 key genes are confirmed (Figure 3(g)).

### 3.3. Analyze Functional Enrichment of Intersection Genes and Construct the PPI Network

**Figure 4:**
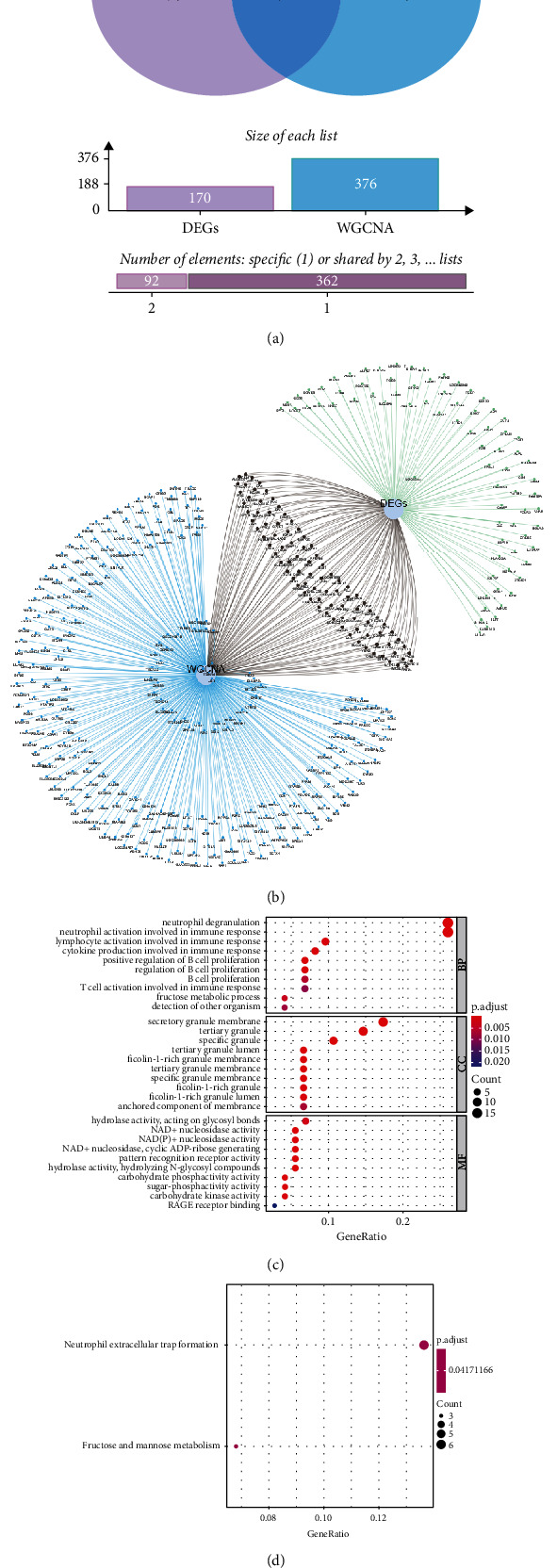
Analyze the functional enrichment of intersection genes. (a) Venn diagram of intersection genes. (b) Network diagram of intersection genes. (c) GO analysis of intersection genes. (d) KEGG analysis of intersection genes.

**Figure 5:**
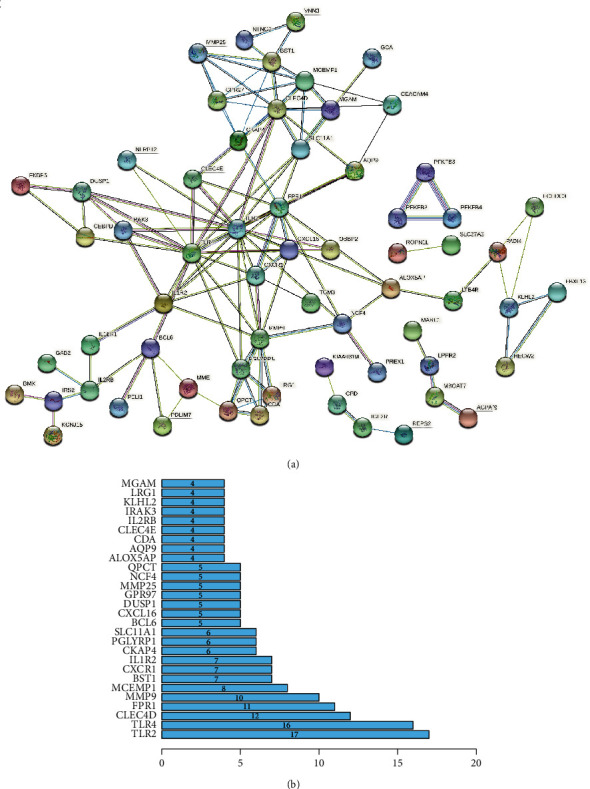
PPI network construction. (a) Construct the PPI network of the intersection genes. (b) Hub gene screen.

Take the intersection of the blue module genes and DEGs, draw the Venn diagram and the network diagram, and obtain 92 intersection genes (Figures 4(a) and 4(b)). Perform GO enrichment analysis (FDR < 0.05) and KEGG enrichment analysis for 92 intersection genes which are concentrated in 30 GO terms (Figure 4(c)) and 2 KEGG pathways including neutrophil extracellular trap formation and fructose and mannose metabolism (Figure 4(d)). Moreover, construct a PPI network and sort the obtained PPI network according to the number of nodes. Select genes with more than 3 nodes and get a total of 28 hub genes (Figures 5(a) and 5(b)).

### 3.4. Screen and Verify the Diagnostic Markers

**Figure 6:**
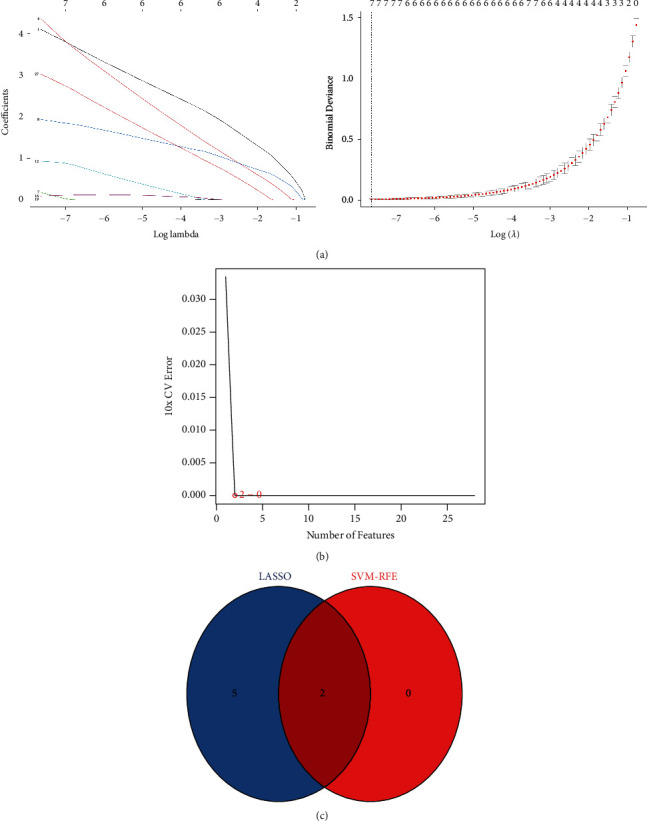
Determined diagnostic markers. (a) Determined diagnostic markers via LASSO. (b) Determined diagnostic markers via the SVM-RFE algorithm. (c) Venn diagram of the intersection of diagnostic markers determined by the two algorithms.

**Figure 7:**
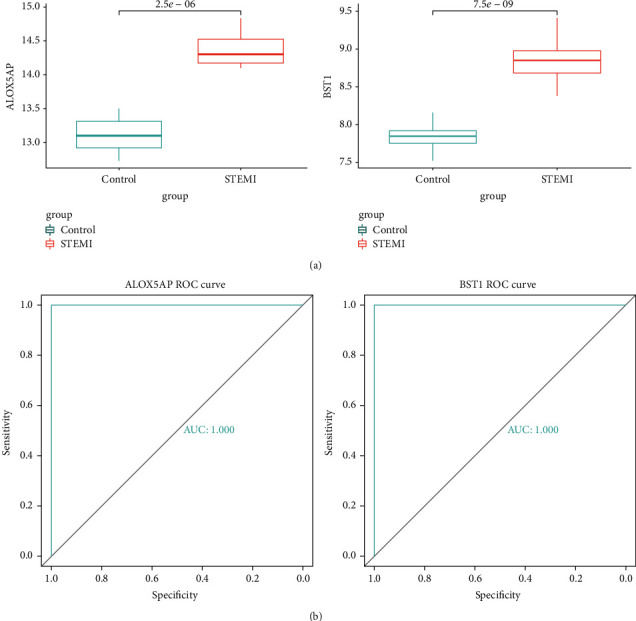
Verification of diagnostic markers. (a) The expression of ALOX5AP and BST1. (b) The ROC curve of ALOX5AP and BST1.

Identify 7 genes from hub genes as diagnostic markers for STEMI by the LASSO logistic regression algorithm (Figure 6(a)); 2 genes as diagnostic markers of STEMI are obtained from hub genes by the SVM-RFE algorithm (Figure 6(b)). Intersect the gene markers got via the two algorithms and finally identify 2 diagnostic markers (Figure 6(c)). Upregulation of ALOX5AP (AUC = 1.00) and BST1 (AUC = 1.00) have a high diagnostic value for STEMI (Figures 7(a) and 7(b)).

### 3.5. Analyze Immune Cell Infiltration

**Figure 8:**
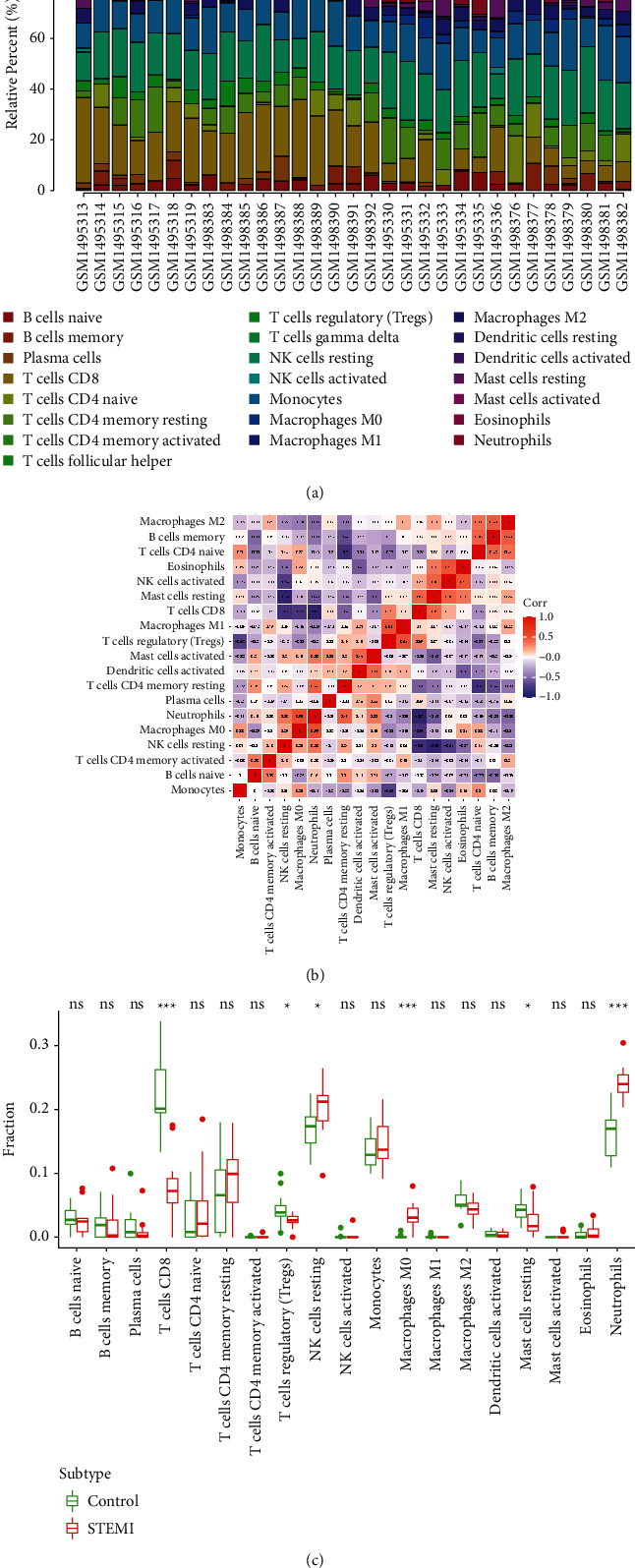
Assessed and visualized immune cell infiltration. (a) Analyzed immune cell infiltration between the STEMI group and control group by CIBERSORT. (b) Heat map of 19 types of immune cells connection. (c) Violin diagram of the proportion of 19 types of immune cells.

The results of immune cell infiltration via CIBERSORT analysis find that there is significant difference in immune cell infiltration between the STEMI group and the control group (Figure 8(a)). Removing the three types of nonexpressing immune cells in the sample, the connection heatmap of the 19 types of immune cells is analyzed (Figure 8(b)). The results show the resting NK cells, M0 macrophages, and neutrophils infiltrated more, while resting CD8+ T cells, Treg cells, and resting mast cells infiltrate less in the STEMI group (Figure 8(c)).

### 3.6. Analyze the Connection between ALOX5AP, BST1, and Infiltrating Immune Cells

**Figure 9:**
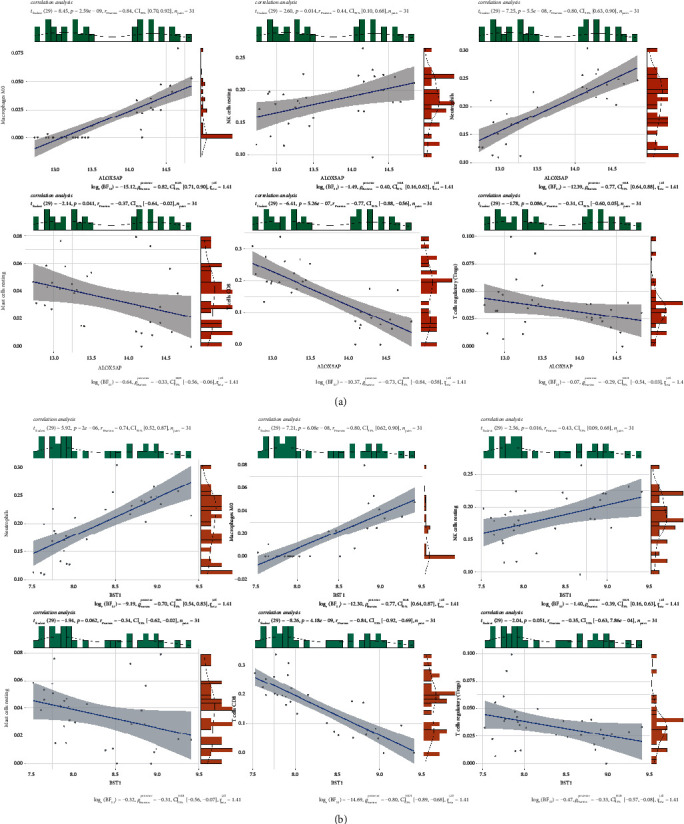
Correlation between ALOX5AP, BST1, and infiltrating immune cells. (a) Connection between ALOX5AP and infiltrating immune cells. (b) Connection between BST1 and infiltrating immune cells.

Connection analysis find that ALOX5AP and BST1 are positively related to resting NK cells, M0 macrophages, and neutrophils, while ALOX5AP and BST1 are negatively related to CD8+T cells, Treg cells, and resting mast cells (Figure 9(a) and Figure 9(b)).

## 4. Discussion

Failure of STEMI patients with PCI to restore an open artery remains as poor outcomes and results in coronary microembolization (CME) [13, 14]. STEMI is an acute coronary syndrome, and inflammation is the primary cause of myocardial injury [15]. Owing to the lack of early diagnostic markers, the STEMI patients lose the great opportunity to treat, resulting in a poor prognosis. Moreover, studies find that immune cell infiltration is related to the development of STEMI [16, 17]. Therefore, obtaining the specific diagnostic biomarkers and studying the immune cell infiltration of STEMI is important to better development of STEMI patients. Bioinformatics provide an effective strategy to screen molecular markers, and CIBERSORT analyses the immune cell infiltration of STEMI. In our study, we define diagnostic biomarkers of STEMI and probe the immune cell infiltration in STEMI.

We obtain the STEMI gene expression data from the GEO database and confirm 710 DEGs and 376 blue module gene which are positively correlated with STEMI. GO-BP enrichment analysis shows that 92 intersection genes between the blue module genes and DEGs are mainly related to neutrophil degranulation and neutrophil and lymphocyte activation related to immune response. 92 intersection genes enrich in neutrophil extracellular trap formation and mannose metabolism. The above findings find that the immune response is related to STEMI. Moreover, there is significant difference in immune cell infiltration between the STEMI group and the control group. Kulasingam et al. [18] found that biomarkers about immune and inflammatory response increase in the pathogenesis of STEMI, which supports the finding of our study.

In our study, ALOX5AP and BST1 are confirmed as diagnostic biomarkers for STEMI via SVM-RFE and LASSO methods. Arachidonate 5-lipoxygenase activating protein (ALOX5AP) controls lipid mediator production to induce macrophage M1 polarization resulting in neutrophilic inflammation [19, 20]. One study showed that ALOX5AP is directly involved in myocardial infarction [21]. SNP rs17216473 of ALOX5AP gene is related to the risk of MI [22]. Bone marrow stromal cell antigen 1 (BST1)/CD157, one of ADP ribosyl cyclase gene family, involves in the regulation of immunoregulatory functions in pathological conditions [23, 24]. A study shows that BST1 could be used as biomarkers of chronic lung allograft dysfunction (CLAD) in bronchoalveolar lavage (BAL) [25]. The urine excretion rates of Ang II-regulated BST1 increase, which is correlated strongly with chronic inflammation [26]. Previous studies show ALOX5AP and BST1 may be related to the progression of STEMI and are the potential diagnostic biomarkers of STEMI, but the clinical study is still needed to confirm the diagnostic value of ALOX5AP and BST1.

To further study the immune cell infiltration in STEMI, use CIBERSORT to confirm the immune infiltration of STEMI. Our study finds that there is an increase of infiltration of resting NK cells, M0 macrophages, and neutrophil, while infiltration of resting CD8+ T cells, Treg cells, and resting mast cells decrease, which may be contacted with the pathogenesis of STEMI. Previous studies found that nonculprit lesions of STEMI patients with high-intensity statin therapy treatment have a decrease in macrophage accumulation [27]. Intracoronary thrombi of STEMI patients show increased infiltration neutrophils [28]. Another study shows Dectin-1 contributes to neutrophil infiltration, which is positively contacted with the severity of cardiac dysfunction of STEMI [7]. The CD8+ T cells in STEMI patients after reperfusion decreased [29]. Galectin-9 inhibits Th17 and upregulates Tregs to inhibit IL-17 production and promote the TGF-*β*1 secretion, resulting in the development of STEMI [30]. Compared to the control samples, the number of Treg decreases in STEMI patients [31]. The above study combined with our study show that resting NK cells, M0 macrophages, neutrophil, resting CD8 T cells, Treg cells, and resting mast cells play an important role in STEMI and should be to further studies.

To analyze the connection between ALOX5AP, BST1, and immune cells, we find that ALOX5AP and BST1 are positively related to resting NK cells, M0 macrophages and neutrophil, while ALOX5AP and BST1 are negative related to CD8+ T cells, Treg cells, and resting mast cells. Studies show that M1 macrophages upregulate the level of ALOX5AP [19, 32]; The hypomethylation of ALOX5AP is strongly related to the neutrophils and dendritic cells (DCs) [33]. A subset of CD3, CD4, CD8 T cells exhibits an increase of BST1 [34]. BST1, a GPI-anchored cell surface glycoprotein, highly expresses in normal monocytes and neutrophils [35, 36]. Peripheral blood NK cells express BST1 [37]. Therefore, we speculate that ALOX5AP and BST1 raise resting NK cells, M0 macrophages, and neutrophil or reduce CD8+ T cells, Treg cells, and resting mast cells to participate in the pathogenesis of STEMI. However, the reliability of our study needs being studied.

In general, we find that ALOX5AP and BST1 are the diagnostic biomarkers of STEMI. We also find that resting NK cells, M0 macrophages, neutrophil, CD8+ T cells, Treg cells, and resting mast cells may be contacted with the development of STEMI. In addition, upregulation of ALOX5AP and BST1 are positively contacted with resting NK cells, M0 macrophages, and neutrophil, while ALOX5AP and BST1 are negatively contacted with CD8+ T cells, Treg cells, and resting mast cells. These immune cells may be related to the pathogenesis of STEMI, providing immunomodulatory therapies for STEMI patients.

This study had several limitations. Clinical data will be needed in future studies. Moreover, functional studies of ALOX5AP and BST1 identified here are needed. Finally, methods based on molecular biology approaches should help validate our findings.

## Data Availability

The data used to support the findings of this study are available from the corresponding author upon request.
